# The Influence of Different Learning Strategies on Pupils’ Learning Motivation: Is Augmented Reality Multimedia Learning Consistent With Traditional Text Learning?

**DOI:** 10.3389/fpsyg.2022.810345

**Published:** 2022-02-25

**Authors:** Xiaojun Zhao, Miaozhuang Liu, Yaqing Liu

**Affiliations:** College of Education, Hebei University, Baoding, China

**Keywords:** AR multimedia learning, text learning, learning strategy, learning motivation, self-generated drawing strategies

## Abstract

How to reduce pupils’ burden and improve learning efficiency? Research shows that non-intelligence factors and learning strategies are the key factors in the effective learning process, and external intervention can play a greater role in these two aspects. The purpose of this study is to explore the effects of different learning strategies on learning motivation under the presentation of augmented reality (AR) multimedia or traditional text learning. Sixty third-grade pupils in Hebei Province were selected, and 2 (learning materials: AR materials and text materials) × 3 (learning strategies: restudying strategies, retrieval practicing strategies, and self-generated drawing strategies) between-subjects design was adopted. The dependent variable is learning motivation, which includes three dimensions: surface motivation, deep motivation and achievement motivation. The results showed that (1) Learning strategies had a significant impact on deep motivation. The deep motivation aroused by retrieval practicing strategies was significantly higher than that aroused by restudying strategies and self-generated drawing strategies. (2) The interaction between learning materials and learning strategies was marginally significant. When learning with AR materials, the achievement motivation of pupils in the restudying strategy group was higher (marginal significant) than that in the retrieval practicing strategy group. Retrieval practicing strategies have positive significance in cultivating deep motivation. The combination of different learning modes and learning strategies will impact achievement motivation.

## Introduction

In recent years, how to reduce pupils’ burden and improve learning efficiency has become a key issue in quality education. Previous studies have confirmed that the psychological structure of efficient learning includes five main internal factors: selective attention, metacognition, learning strategies, nonintellectual factors and implicit cognition (Shen and Bai, 2006). Studies have shown that non-intelligence factors are the driving force of efficient learning, while learning strategies provide guarantee for efficient learning process. Improving the level of both will improve students’ learning efficiency. At the same time, external intervention can play a greater role in these two aspects (Shen and Bai, 2006). Lu (2007) also proposed that scientific learning methods, appropriate use of learning strategies and fun in learning are the three basic characteristics of students’ efficient learning. In other words, studying how to combine specific material content with learning strategies to promote students’ learning motivation is an important topic in efficient learning. Previous studies have proven that learning strategies, teaching environment and learning motivation affect the learning effect and interact with each other.

The psychological state of children in primary school is at a critical stage of development. In late childhood, children’s thinking transitions from concrete image to abstract logical thinking. At this time, children’s internal language is also developing, but it cannot be as perfect as adults, and still has great concrete image. There is a critical age in the transition stage, approximately 9–11 years old, when dialectical thinking begins to emerge. The second or third grade is the key period for the development of pupils’ written language. Before that, the written language level lags behind the oral language level, while the written language gradually shows advantages after the fourth grade. As for children’s learning motivation, generally speaking, the lower the grade, the more specific their learning motivation is. With the growth of grade, their learning interest has gradually shifted from external activities, such as games, to internal learning content (Lin, 2009). However, this change is not necessarily formed naturally. According to the viewpoint of evolutionary psychology, children’s natural interest lies in exploring the environment and social interpersonal relationships, which conflicts with the motivation to master learning ability. In other words, the development of students’ learning motivation in the critical period of development needs external forces, such as family and school education, to guide and intervene (Wang and Tan, 2014).

Stimulating and maintaining learning motivation is a teaching focus in primary school. Motivation is the internal driving force to stimulate individual action and guide it to a certain goal. Therefore, learning motivation can be defined as the internal motivation for students to learn spontaneously, which is a key factor affecting students’ academic performance (Lin et al., 2003; Steinmayr and Spinath, 2009). Biggs (1987) divided learning motivation into three dimensions: surface motivation (SM), deep motivation (DM), and achievement motivation (AM). Surface motivation refers to the motivation to learn due to surface material stimuli, such as rewards. Students with high surface motivation usually take coping with exams or classroom tests as their learning goals. Deep motivation refers to the motivation to learn due to deeper non-material stimuli, such as mastering knowledge and acquiring skills, which originates from the real interest in the learned content. Achievement motivation refers to an individual’s drive to achieve goals and achieve success (Zhang and Yang, 1999; Jia et al., 2012). The impact of learning motivation on learning performance is significant. Learning motivation drives students to conduct learning activities. The motivation pointing results of different dimensions are different; that is, there are differences in learning objectives, so the efforts and actions are not consistent (Zhang and Shen, 2005).

Learning strategies are also an important dimension affecting learning efficiency. There are many types of learning strategies, and the most commonly used strategies in the classroom are restudying strategies and retrieval practicing strategies. Tests are a form of retrieval practicing strategy. In daily learning activities, students consciously use restudying strategies for learning, but by adding retrieval practice between learning and a final test, students’ learning effect is significantly better than pure repetition learning (Roediger and Karpicke, 2006; Karpicke and Roediger, 2008). Retrieval practicing strategies are helpful to maintain long-term memory so as to improve the learning effect. In recent years, the self-generated drawing strategy with high popularity has to be mentioned. The self-generated drawing strategy refers to students expressing what they have learned through drawing when learning content without pictures, that is, learning content visualization. The key points of the self-generated drawing strategy are self-generation and drawing representation. According to the Generative Theory of Drawing Construction (GTDC), this process requires students to carry out some constructive activities. In this process, learners will select, organize and integrate materials, and self-monitoring and regulation will also be activated, so that learners can more deeply understand the text content and help master knowledge (van Meter and Garner, 2005; Wang et al., 2019).

With the development of science and technology, multimedia technology is also being updated and iteratively applied to learning activities. The new learning method based on AR enhancement technology has good interaction and brings learners a completely different experience from the traditional learning method. The basic characteristics of AR technology mainly include three points: the integration of virtual and real environment, real-time interaction and 3D registration. Augmented reality (AR) can make users experience a strong sense of authenticity and presence. In educational psychology, the impact of the application of AR technology on teachers’ instructional design and students’ cognitive strategies is an important direction of scientific research (Zhao et al., 2014; Zhou et al., 2015). AR (augmented reality) technology can realize the transformation of students’ knowledge from abstract to concrete, display the text content in a concrete and realistic way, make the text content easier to understand and enhance interest, and compensate for the shortcomings of traditional learning methods (Liu and Chen, 2019). The benefits of applying augmented reality technology to teaching activities have been studied in recent years. Students learn through AR materials, and the visualization effect generated by 3D model greatly enhances students’ understanding and perception of the learned abstract concepts. In addition, the presence and interactivity brought by AR materials make students feel like they are on the scene, which can improve students’ sense of existence and concentration (Cai et al., 2016).

According to previous studies, learning motivation, learning strategies, and the types of learning materials can affect the learning effect; and previous studies have stated that there is a certain correlation between learning strategies and learning motivation. The two promote each other. Students with strong learning motivation will use more meta learning strategies, and the use of learning strategies will also enhance students’ learning motivation (Sun and Meng, 2021). However, research on the relationship between the three is still relatively rare. Therefore, this study is expected to explore the influence of different types of learning modes and learning strategies on learning motivation, and use the experimental method to study whether the three learning strategies of restudying, retrieval practicing, and self-generated drawing have different effects on pupils’ learning motivation under the presentation of two different learning materials of AR multimedia and traditional text. The possible reasons for the existence of different effects also will be discussed.

## Methods

### Participants

Sixty pupils in grade 3 were recruited by cluster random sampling from one public primary school in Hebei Province, China. The gender ratio was balanced (27 girls and 33 boys). The age range was 8–11 years old (two pupils aged 8, 38 pupils aged 9, 19 pupils aged 10, one pupil aged 11), and the average age was 9.32 years old (SD = 0.57). All subjects were native speakers of Chinese and had normal vision or corrected vision. They had not participated in similar experiments before and voluntarily participated in this study.

### Materials

#### Vocabulary Test Questions

The 10 English words used in the experiment were compiled into vocabulary test questions. The subjects were asked to review whether they had learned the corresponding English words according to the Chinese words provided, score the words they had not learned “×” and tick “√” and write the English spelling of the words they had learned. In order to ensure that the subjects had the same initial level of learning, those pupils who could write English spelling by dictation were eliminated.

#### Augmented Reality Materials

**Figure 1:**
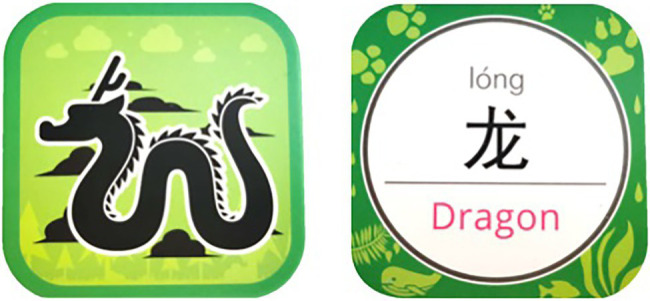
The front and back of augmented reality (AR) cognitive card.

**Figure 2:**
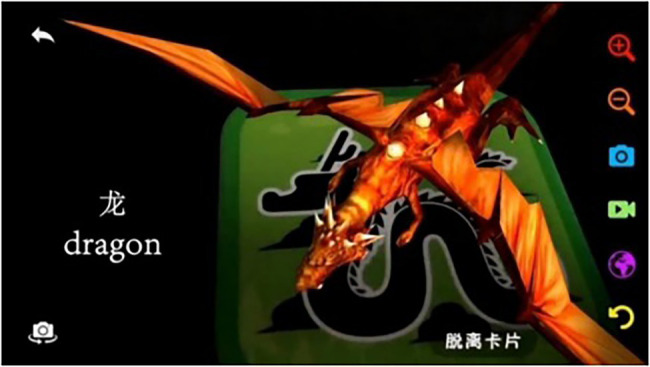
Augmented reality video presentation.

Ten AR cognitive cards, the front and back of which are shown in Figure 1, were selected. Each card corresponds to a type of animal. The animal was scanned with AR software, and the screen presented a color 3D image of the corresponding animal equipped with an English teaching voice, thus making full use of the interactivity of AR technology. In the experiment, the AR cognitive card was scanned and made into an AR video presentation. The video content includes 3D dynamic animal images, Chinese and English words and English teaching pronunciations (as shown in Figure 2).

#### Text Materials

The participants used the text materials made by the researchers and followed the teaching textbooks. The learning content was the same 10 Chinese and English words as with the AR materials and was presented in the form of text, including 2D static animal images and Chinese and English words. In order to simulate traditional text learning conditions, pronunciation is not taught.

#### Pupils’ Learning Motivation Questionnaire

This questionnaire was revised by Jia et al. (2012), and the original questionnaire was adapted from the Learning Process Questionnaire of Biggs (1987). The content includes three motivation dimensions: surface type, deep type, and achievement type. This questionnaire consists of 16 questions and uses four-point scoring. The revised questionnaire has good reliability and validity, the correlation coefficient between each subscale is low, and the correlation coefficient with the total scale is high, which represents good structural validity.

#### Display Equipment

A 15.6-inch laptop with the screen resolution set to 1920 × 1080 was used.

### Design

A 2 (learning materials: AR materials and text materials) × 3 (learning strategies: restudying strategies, retrieval practicing strategies, and self-generated drawing strategies) between-subjects design was adopted, and the dependent variable was learning motivation.

### Procedure

Firstly, the subjects were randomly divided into six groups, text restudying group, text retrieval practicing group, text drawing group, AR restudying group, AR retrieval practicing group and AR drawing group, so as to ensure the randomness of the learning materials and learning strategies used, with 10 people in each group. All subjects were screened using vocabulary test questions. In order to ensure the same initial level, subjects who could dictate any English word were excluded.

Secondly, in the learning stage, the subjects in each group learned the corresponding materials first, the text group learned according to the distributed text materials, and the AR group learned according to the AR video materials for 80 s. After learning, the text repetition group and AR repetition group repeated learning, that is, they repeated the learned content twice for 80 s each time. The text extraction group and AR extraction group randomly extracted the learned content. That is, Chinese clue words were presented in the text or screen; the subjects were asked to recall the corresponding English words, such as “Peacock -?”; and each word was addressed for 16 s. The text drawing group and the AR drawing group conducted self-generated drawing. That is, the text prompts were combined with the materials learned above to generate corresponding images in the mind and those images were drawn. An example is the following: “A dragon is a mythical animal and can spit fire. Its body is like a lion and its head is like a horse. Its two huge wings are like bats.” The subjects drew by themselves according to the requirements, and the time was 160 s.

Finally, all subjects completed the pupils’ learning motivation questionnaire according to the same guidance. The questionnaire includes three dimensions, surface motivation, deep motivation and achievement motivation; and collected learners’ learning motivation level data under different conditions. In the measurement results, the total scores of all items are the indicators to measure the overall level of learning motivation, and the total scores of each dimension are the indicators to measure the level of different sub-types of learning motivation.

### Data Processing

The experimental data were processed and analyzed using SPSS 26.0. For the abnormal data, the box chart is used to filter the abnormal values, and the group average value is used for replacement processing. When processing the data, firstly, the learning motivation under different learning modes is described and statistically analyzed. Secondly, multivariate analysis of variance was used to test the main effects of learning materials and learning strategies on learning motivation and their interaction effects.

## Results

The learning motivation of grade 3 pupils when using different learning materials and learning strategies was described and counted. The specific results were shown in Tables 1, 2.

**Table 1:** Descriptive statistics of learning motivation under different learning materials.

	AR (*M* ± **SD**)	Text (*M* ± **SD**)	*t*	*d*
Surface motivation	8.27 ± 1.87	7.37 ± 2.30	1.66	0.429
Deep motivation	26.13 ± 3.79	27.37 ± 3.15	−1.37	−0.356
Achievement motivation	12.70 ± 3.42	12.30 ± 3.64	0.44	0.113
Total learning motivation	47.10 ± 6.77	47.03 ± 6.45	0.04	0.011

* *p* < 0.05,

** *p* < 0.01,

*** *p* < 0.001.

**Table 2:** Descriptive statistics of learning motivation under different learning strategies.

	Restudying strategies (*M* ± **SD**)	Retrieval practicing strategies (*M* ± **SD**)	Self-generated drawing strategies (*M* ± **SD**)	*F*	$\eta^{2}$
Surface motivation	7.45 ± 2.06	8.15 ± 1.87	7.85 ± 2.46	0.537	0.018
Deep motivation	26.20 ± 3.21	28.95 ± 2.35	25.10 ± 3.77	7.866^**^	0.216
Achievement motivation	12.45 ± 3.98	12.35 ± 3.34	12.70 ± 3.33	0.051	0.002
Total learning motivation	46.10 ± 6.50	49.45 ± 5.86	45.65 ± 6.92	2.079	0.068

* *p* < 0.05,

** *p* < 0.01,

*** *p* < 0.001.

Taking learning materials and learning strategies as independent variables and deep motivation as dependent variables, multivariate analysis of variance was carried out. The results showed that the main effect of learning strategies was significant [*F*(2, 54) = 8.313, *p* < 0.01, $\eta_{p}^{2}$=0.235]. Furthermore, pupils’ deep motivation using retrieval practicing strategies (*M* = 28.95, SD = 2.35) was the highest, which was significantly higher than when using restudying strategies (*M* = 26.20, SD = 3.21), *p* < 0.01, and self-generated drawing strategies (*M* = 25.10, SD = 3.77), *p* < 0.001.

**Figure 3:**
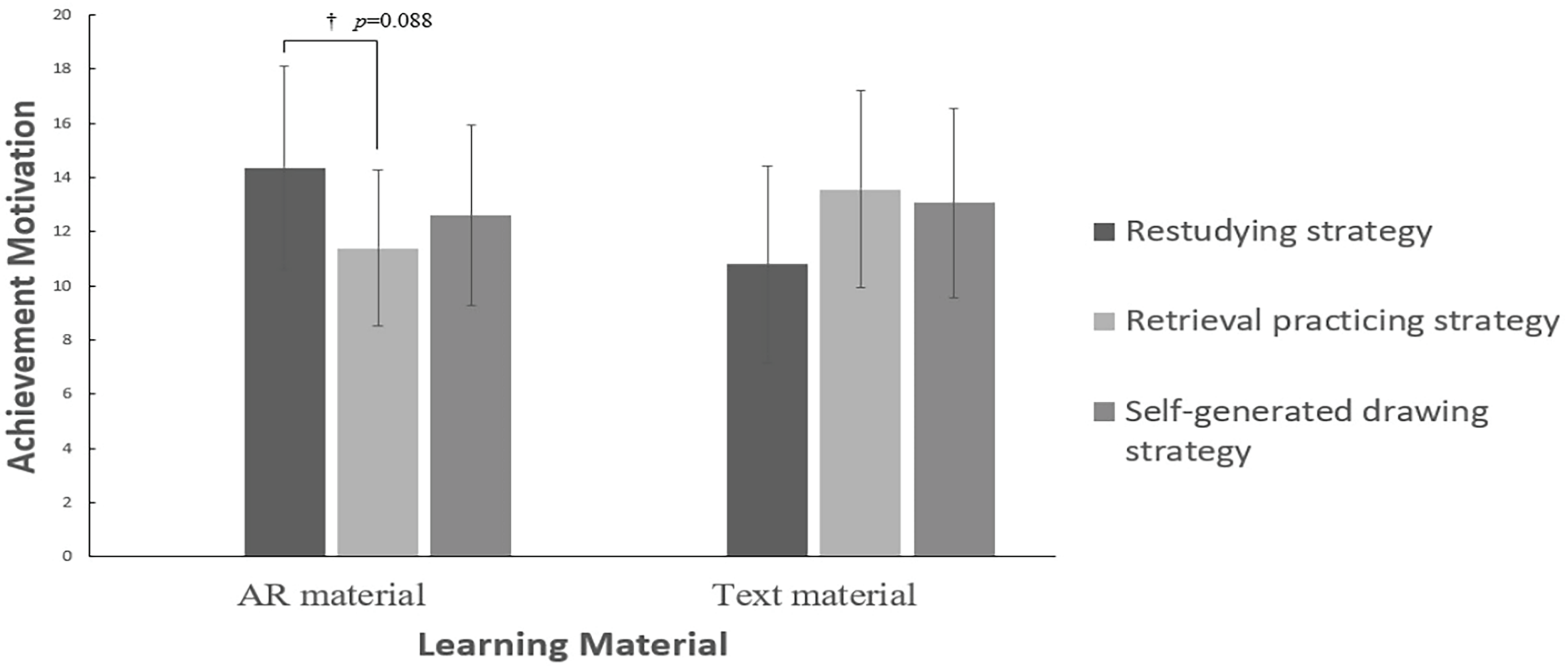
Achievement motivation under different learning conditions. ^*^p < 0.05, ^**^p < 0.01, ^***^p < 0.001, ^†^0.05 < *p* < 0.10.

Taking learning materials and learning strategies as independent variables and achievement motivation as dependent variables, multivariate analysis of variance was carried out. The results showed that the interaction between learning materials and learning strategies was marginally significant [*F*(2, 54) = 2.916, 0.05 < *p* < 0.10, $\eta_{p}^{2}$=0.097]. The simple effect analysis (Figure 3) showed that when learning with AR materials, the students’ achievement motivation in the restudying strategy group (*M* = 14.10, SD = 3.76) was higher than (marginal significant) that in the retrieval practicing strategy group (*M* = 11.40, SD = 2.88).

## Discussion

### Significance of Learning Strategies for Cultivating Pupils’ Deep Motivation

The use of learning strategies is closely related to students’ learning motivation (Zhang and Guo, 2001; Zhou et al., 2005). Compared with restudying strategies, retrieval practicing strategies have obvious advantages in promoting meaningful learning. They can trigger the deep processing of learning materials and encourage students to form the goal orientation of mastering knowledge and skills (Ma et al., 2014). Deep processing strategies can effectively improve pupils’ perceived ease of use of learning content. That is, deep processing strategies affect students’ cognition of learning ease, reduce their learning frustration, make them think actively and explore actively, and have a good effect on cultivating deep motivation (Zhang and Yang, 1999; Liu et al., 2015). The deep motivation of self-generated drawing strategy in text material learning is higher than that in AR material. According to cognitive load theory, the increase of cognitive load will hinder the individual information processing process, and the size of internal cognitive load (ICL) is related to the complexity of the material itself. Self-generated drawing is mainly a process in which individuals form psychological images by word processing, and draw them. Compared with the plane images presented by text materials, the processing complexity of 3D dynamic images presented by AR materials increases, and its ICL also increases, which may reduce students’ learning efficiency and further lead to the reduction of deep motivation (Ding and Luo, 2009; Sweller et al., 2011). Cao et al. (2005) also pointed out that the correct rate of working memory resource strategy used by grade 3 primary school students will decrease significantly with the increase of cognitive load difficulty. Therefore, the disadvantages of the application of AR technology in education also need to be considered. The learning strategies discussed in this study are mainly based on visual presentation. Previous studies on multimedia learning pointed out that learning strategies from the perspective of hearing will also affect learners’ learning process and results. Xiong et al. (2018) proved through experiments that when presenting multimedia learning materials, reducing the pitch by 0.5 ERB can effectively induce learners’ positive emotions. Mayer (2009) also proposed that compared with a single visual channel material, the presentation of audio-visual dual channel materials in multimedia learning is conducive to learners’ memory and transfer, that is, to produce a Modality Effect. Are the research results in the field of multimedia learning consistent with those in the field of AR learning? Future research can further explore the impact of learning strategies on children’s learning motivation in AR learning based on multi-sensory channels such as hearing and touch.

### The Combination of Different Learning Modes and Learning Strategies Leads to a Change in Pupils’ Achievement Motivation

As can be seen from Figure 3, when learning with text materials, students’ achievement motivation of using extraction practice strategy is higher than that of repeated learning strategy. Perhaps because the sample size does not reach the ideal level, the significance of this trend can not be verified, which needs to be tested by expanding the sample in follow-up research. According to the theory of achievement motivation, when the probability of success is equal, the attraction of goals is the main factor affecting individual achievement motivation (Jia and Liu, 2011; Anderman, 2020). When learning with text materials, compared with retrieval practicing strategies, restudying strategies are more boring, consumes pupils’ passion for learning and may reduce pupils’ achievement motivation. AR materials are presented in 3D. Although the materials appear repeatedly during learning, pupils can still maintain a high degree of attention. However, the presentation time of 3D animation in the retrieval practicing strategy is short, and the interest cannot be sustained, so pupils’ achievement motivation is relatively low. Among the students who adopt restudying strategies, the achievement motivation when using AR materials is higher than that when using text materials, which also proves that presenting learning materials in the form of AR video can effectively stimulate students’ achievement motivation and then help to improve their academic performance (Schüler et al., 2021).

Therefore, in order to cultivate pupils’ positive learning motivation in primary school, teachers need to guide pupils to adopt diversified learning strategies to transform different learning contents of different disciplines. For the learning materials that need to be understood and explored, the effect of using retrieval practicing strategies will be more prominent. Finally, the application of AR multimedia technology in the field of education can process boring text materials and present them in the form of animation images, which is helpful to improve pupils’ achievement motivation.

## Conclusion

(1) Retrieval practicing strategies have positive significance in cultivating deep motivation. (2) The combination of different learning modes and learning strategies will impact achievement motivation.

## Data Availability Statement

The raw data supporting the conclusions of this article will be made available by the authors, without undue reservation.

## Ethics Statement

The studies involving human participants were reviewed and approved by the academic and ethics committee of school of education in Hebei University. The participants provided their written informed consent to participate in this study. Written informed consent was obtained from the individual(s) for the publication of any potentially identifiable images or data included in this article.

## Author Contributions

XZ: conceived the study. XZ, ML, and YL: carried out the study and wrote the paper. All authors contributed to the article and approved the submitted version.

## Funding

This study was supported from Education General Project of National Social Science Foundation of China in 2020 “The Research on Cognitive and Emotional Mechanism of Augmented Reality (AR) Multimedia Learning and Its Promotion to Primary School Students’ Efficient Learning” (Project approval number: BBA200031).

## Conflict of Interest

The authors declare that the research was conducted in the absence of any commercial or financial relationships that could be construed as a potential conflict of interest.

## Publisher’s Note

All claims expressed in this article are solely those of the authors and do not necessarily represent those of their affiliated organizations, or those of the publisher, the editors and the reviewers. Any product that may be evaluated in this article, or claim that may be made by its manufacturer, is not guaranteed or endorsed by the publisher.
